# Verification of threshold for image intensity ratio analyses of late gadolinium enhancement magnetic resonance imaging of left atrial fibrosis in 1.5T scans

**DOI:** 10.1007/s10554-019-01728-0

**Published:** 2019-11-20

**Authors:** Litten Bertelsen, Francisco Alarcón, Laura Andreasen, Eva Benito, Morten Salling Olesen, Niels Vejlstrup, Lluis Mont, Jesper Hastrup Svendsen

**Affiliations:** 1 Department of Cardiology, Centre for Cardiac-, Vascular-, Pulmonary and Infectious Diseases, Rigshospitalet, University of Copenhagen, Copenhagen, Denmark; 2 Department of Cardiology, Unitat de Fibril.lació Auricular (UFA) Hospital Clinic, University of Barcelona, Barcelona, Spain; 3 Institut d’Investigacions Biomédiques August Pi i Sunyer (IDIBAPS), Barcelona, Spain; 4 Department of Biomedical Sciences, University of Copenhagen, Copenhagen, Denmark; 5 Department of Clinical Medicine, Faculty of Health and Medical Sciences, University of Copenhagen, Copenhagen, Denmark

**Keywords:** Cardiac magnetic resonance imaging, Left atrial late gadolinium enhancement, Image intensity ratio, Atrial fibroses, Atrial fibrillation

## Abstract

The use of cardiovascular magnetic resonance imaging left atrial late gadolinium enhancement (LA LGE) is increasing for fibrosis evaluation though the use is still limited to specialized centres due to complex image acquisition and lack of consensus on image analyses. Analysis of LA LGE with image intensity ratio (IIR) (pixel intensity of atrial wall normalized by blood pool intensity) provides an objective method to obtain quantitative data on atrial fibrosis. A threshold between healthy myocardium and fibrosis of 1.2 has previously been established in 3T scans. The aim of the study was to reaffirm this threshold in 1.5T scans. LA LGE was performed using a 1.5T magnetic resonance scanner on: 11 lone-AF patients, 11 age-matched healthy volunteers (aged 27–44) and 11 elderly patients without known history of AF but varying degrees of comorbidities. Mean values of IIR for all healthy volunteers +2SD were set as upper limit of normality and was reproduced to 1.21 and the original IIR-threshold of 1.20 was maintained. The degree of fibrosis in lone-AF patients [median 9.0% (IQR 3.9–12.0)] was higher than in healthy volunteers [2.8% (1.3–8.3)] and even higher in elderly non-AF [20.1% (10.2–35.8), p = 0.001]. The previously established IIR-threshold of 1.2 was reaffirmed in 1.5T LA LGE scans. Patients with lone AF presented with increased degrees of atrial fibrosis compared to healthy volunteers in the same age-range. Elderly patients with no history of AF showed significantly higher degrees of fibrosis compared to both groups with younger individuals.

**Electronic supplementary material:**

The online version of this article (10.1007/s10554-019-01728-0) contains supplementary material, which is available to authorized users.

## Introduction

The clinical demand for cardiac magnetic resonance imaging (CMR) and the corresponding scientific developments have been expanding during the past 20 years [1]. Late gadolinium enhancement is today a well-established method for detecting fibrosis in the ventricular myocardium [2]. Since its introduction in 2007 [3], CMR imaging of left atrial fibrosis with late gadolinium enhancement (LA LGE) has received increasing interest [4–9]. The method has proven valuable in detection of pre-ablation atrial fibrosis [4, 8, 10–13] but also in evaluation of ablation procedures and detection of possible gaps in ablation lines after pulmonary vein isolation [10, 12, 14–16].

Despite its many promising possibilities, LA LGE has remained a method reserved for highly specialized centres and previous studies have reported both positive [4, 8, 14, 15] as well as disappointing results [5, 7, 16–19]. The clinical adoption of LA LGE has been slowed by complex image acquisition protocols reflected in high exclusion rates of scans in clinical studies (9–32%) [4, 5, 8, 9, 13, 20] and furthermore of a lack of consensus on an objective image analysis method [21]. Initially, fibrotic areas were evaluated visually [3] but the need for an objective analysis method resulted in development of various reference-based analysis methods. Different anatomic regions have been used as reference; healthy atrial myocardium [11], nulled ventricular myocardium [19], fibrotic atrial myocardium [14], blood pool variability [12, 22] or blood intensity [23]. All methods aim to distinguish between healthy and fibrotic atrial myocardium. As the interstitial fibrosis prior to ablation in otherwise similar atrial fibrillation (AF) patient groups vary from 6 to 31% [4, 5, 12, 18, 20, 24–27] and in healthy volunteers from 1.7 to 8.9% [11, 27] with various analysis methods, there is an obvious need for an objective analysis method for continuous development of LA LGE and for obtaining trustworthy clinical results.

The image intensity ratio (IIR) analysis method of LA LGE uses the mean blood volume intensity as reference [23, 25]. By normalization with the mean blood pool intensity the variability due to timing and dose of contrast, renal function, coil proximity, and haematocrit is reduced and the IIR analysis method hence provides an objective assessment of LA LGE. The IIR analysis method was initially introduced by Khurram et al. who defined IIR thresholds based on electroanatomic mapping (EAM) [23]. The IIR analysis method has shown good consistency and absolute agreement when compared to other thresholding methods [6]. Based on data from healthy volunteers Benito et al. found an upper IIR threshold of 1.2 for healthy atrial myocardium [25]. The study by Benito et al. was performed on 3 Tesla (T) magnetic resonance (MRI) scanners and so far, this threshold has not been validated or reproduced at 1.5T. Currently 1.5T scanners are more widely used than 3T scanners for cardiac MRI. Although available data suggest that the field strengths provide similar results [6], differences in signal-to-noise and contrast-to-noise between the two field strengths may result in differences in measured tissue intensities [28]. This means that a validation of the thresholds in both field strengths is important before using the thresholds in large scale studies or clinical work [25].

The aim of this study was to reproduce the LA LGE IIR threshold found at 3T for healthy atrial myocardium in healthy volunteers scanned on a 1.5T MRI scanner.

## Methods

### Study population

A total of 33 participants were included for the study: 11 healthy volunteers, 11 patients with lone paroxysmal atrial fibrillation (AF) and 11 elderly patients.

The group of healthy volunteers served as reference for healthy tissue and were used for calculating the image intensity ratio (IIR) threshold at 1.5T. This group consisted of 11 healthy volunteers, age-matched to the below-mentioned lone AF patients. The healthy volunteers had no history of heart disease or other health conditions and did not receive prescription medication.

Two patient groups were included for verification of the threshold obtained: 11 patients with lone paroxysmal atrial fibrillation aged less than 45 years of age and no prior ablations and 11 elderly patients (minimum 70 years of age) with no history of AF but with various degrees of known diabetes mellitus, hypertension, congestive heart disease and/or previous stroke.

Exclusion criteria for all included participants were glomerular filtration rate < 60 mL/min, claustrophobia, known gadolinium allergy and implanted ferromagnetic metals.

All included participants were scanned for research purposes and provided written informed consent prior to inclusion. The study protocol conforms to the ethical guidelines of the 1975 Declaration of Helsinki as reflected in a priori approval by the local ethics committee of the Capital Region of Denmark (Protocol Number H-1-2011-044 and H-4-2013-025).

### Image acquisition

A 1.5T MRI scanner (Aera, Siemens Healthcare, Germany) with an 18-channel body coil was used to scan all included patients and healthy volunteers at Rigshospitalet, Copenhagen, Denmark. After scout sequences, long axis cine images (two-chamber, three-chamber and four-chamber) images were acquired for planning of a short axis stack and aid in delineation of chambers. An axial cine stack ranging from the basis to the aortic arch and a short axis cine stack covering the entire left ventricle were obtained for measurement of left atrial and left ventricular volumes, respectively (steady-state free precession cine sequences (8 mm; 2 mm gap; 25 phases; field of view (320–360) × 360 adjusted for each patient; matrix size [182–224) × (138–224)] at 10–15 s end-expiratory breath-holds).

The left atrial late gadolinium enhancement (LA LGE) scan was performed 20 min after bolus injection of 0.2 mmol/kg gadobutrol (Gadovist, Bayer, Berlin, Germany), with a maximum of 15 mmol in total [6]. The LA LGE scan consisted of a free-breathing respiration-navigator-gated 3D FLASH sequence with FatSat and ECG-gating (atrial end-diastole, determined from four chamber cine). Typical parameters were TR/TE 4.67/1.94 ms and bandwidth of 300 Hz. Inversion time was set according to a scout-sequence in a mid-ventricular image (270–310 ms). Flip angle was 20° and slice thickness 1.5 mm. Pixel spacing was 0.70 × 0.70 mm. No parallel-imaging was used. The mean acquisition time was 9 min 12 s ranging from 3 min 12 s to 16 min 27 s.

### Image analyses

Volumetric measurements were performed in CVI^42^ (v. 5.6.6, Circle Cardiovascular Imaging Inc., Calgary, Canada). On short axis cine images left ventricular (LV) end-diastolic and end-systolic phases were traced manually at the endo- and epicardial border. Left ventricular outflow tract (LVOT) was included in the blood pool; papillary muscles were excluded, using windowing for the endocardial border detection. Epicardium was delineated in both phases to compare left myocardial mass in end-diastole and end-systole. Left atrial (LA) volumes were traced manually on axial cine images. Left atrial appendage was included in left atrial volume. Minimum and maximum volumes were indexed to body surface area.

**Fig. 1:**
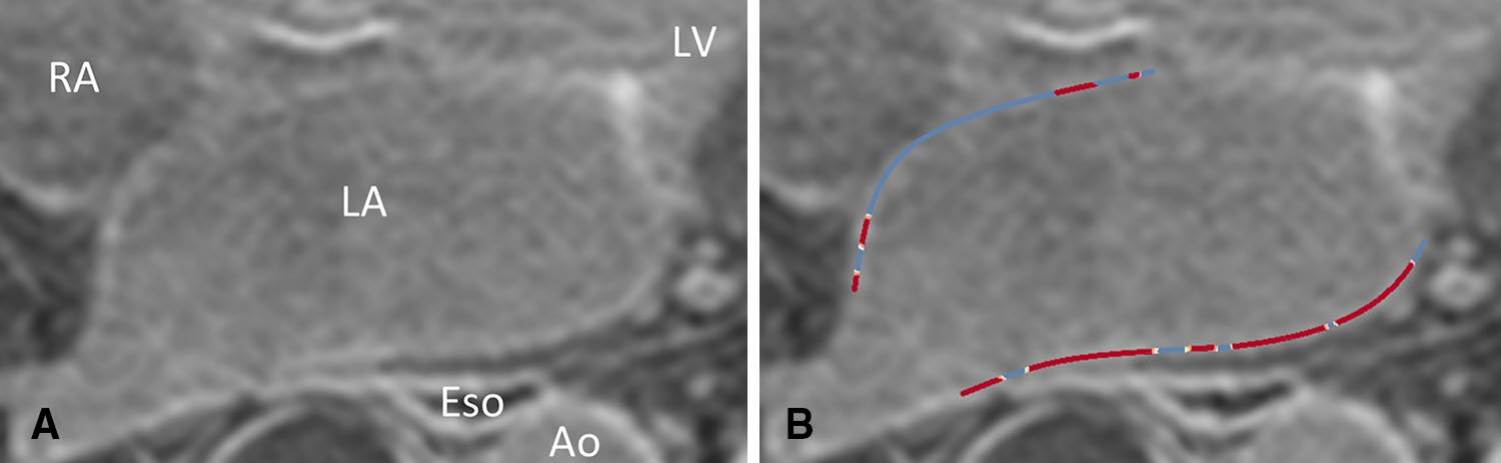
Example of left atrial late gadolinium enhancement image. **A** original image. Blood intensity is 60 and image intensity ratio threshold hence (60 × 1.2 =) 72. **B** depicts segmented atrial wall, blue indicating atrial wall below threshold, red indicating atrial wall above threshold. *RA* right atrium, *LA* left atrium, *Eso* esophagus, *LV* left ventricle, *Ao* aorta

LA LGE scans were analysed using ADAS ^®^ image post-processing software (Galgo Medical SL, Barcelona, Spain). On all axial images the atrial blood pool was initially segmented and atrial wall was interpolated automatically into a 3D shell, which was manually adjusted. To avoid epi- or endocardial artefacts or partial volume effects, the software was set to create a single mid-myocardial layer, which was manually adjusted according to MRI images to represent mid-atrial wall. Inflow artefacts were excluded. Pulmonary veins and mitral valves were excluded for fibrosis analyses. Pixel intensities were calculated automatically and shown on the 3D shell. Atrial wall pixel intensities and mean blood pool intensity were exported from ADAS ^®^. Image intensity ratio (IIR) values of all atrial wall pixels were calculated as atrial wall pixel intensity divided by mean blood pool intensity) (see Fig. 1).

### Image intensity ratio threshold

The threshold sought for was the discrimination between healthy and fibrotic atrial tissue. In accordance with Benito et al. [25], normal LA IIR was extracted from healthy volunteer scans. All IIR values from healthy volunteers were plotted in a histogram and the upper limit of normality (i.e. the threshold value for normal atrial myocardium vs. fibrotic myocardium) was defined as the mean IIR value + 2 SDs.

Histograms of IIR-values for the two remaining groups were constructed and all three histograms were tested for skewness. Skewness-values close to zero represent symmetry, negative values represent left-tailed distributions and increasingly positive values represent increasingly right-tailed distributions. A right-tailed distribution would in this case represent increased degrees of fibrosis.

To investigate whether there was possible age-related fibrosis in the healthy control group we divided the group into a younger and older half based on median age and compared the thresholds in these two groups.

### Detection of different degrees of native fibrosis

The obtained threshold distinguishing between healthy and fibrotic atrial wall was subsequently applied to the segmentations performed in ADAS ^®^ to detect the degree of native fibrosis in the atrial wall in the three included groups. The degree of fibrosis was calculated as the percentage of pixels in the atrial wall above the set threshold.

### Statistics

Continuous variables are presented as mean ± SD or median (interquartile range) and compared with one-way ANOVA/Kruskal–Wallis test. For pairwise comparisons, *t*-tests were performed. Non-normal distribution variables were logarithmically transformed and tested for normality prior to statistical tests. Inter- and intra-observer variability was performed in a subset of 15 randomly selected participants (five from each group). Intraclass correlation coefficient (ICC) was evaluated (good correlation was defined as ICC > 0.70). Bland–Altman plots were constructed and visually inspected for bias or proportional error. P-values < 0.05 were considered statistically significant. All analyses were performed in SPSS Statistics (version 22, International Business Machines, Armonk, New York, USA).

## Results

Table 1 presents clinical baseline data for all included participants. Healthy volunteers and lone AF patients were of similar age (37 SD 6 vs. 39 SD 5 years) and patients in the elderly non-AF group were 76 SD 5 years. None of the healthy volunteers or lone-AF patients had any history of hypertension, diabetes mellitus, congestive heart disease or previous stroke, whereas these conditions were present to a varying degree in the elderly group (18–82%).

**Table 1:** Baseline data on all included volunteers and patients

	Healthy volunteers n = 11	Lone AF n = 11	Elderly non-AF n = 11
Age (years)	37 ± 6	39 ± 5	76 ± 5
Male (%)	9 (82%)	10 (91%)	6 (55%)
Body surface area (m^2^)	2.0 ± 0.1	2.1 ± 0.2	1.9 ± 0.2
Hypertension	0	0	9 (82%)
Diabetes mellitus	0	0	5 (45%)
Congestive heart disease	0	0	2 (18%)
Previous stroke/TIA	0	0	4 (36%)
Medication			
Beta blockers	0	0	1 (9%)
Diuretics	0	0	5 (45%)
Other antihypertensives	0	0	9 (82%)
Antidiabetics	0	0	5 (45%)
Statins	0	0	9 (82%)
ASA and/or platelet-inhibitors	0	0	7 (64%)

*AF* atrial fibrillation, *TIA* transitory ischemic attack, other antihypertensives include ACE-inhibitors, angiotensin II receptor antagonists and calcium antagonists, *ASA* acetylsalicylic acid

Values are given as mean ± 1 SD or absolute numbers (% of total)

### Image intensity ratio threshold

**Fig. 2:**
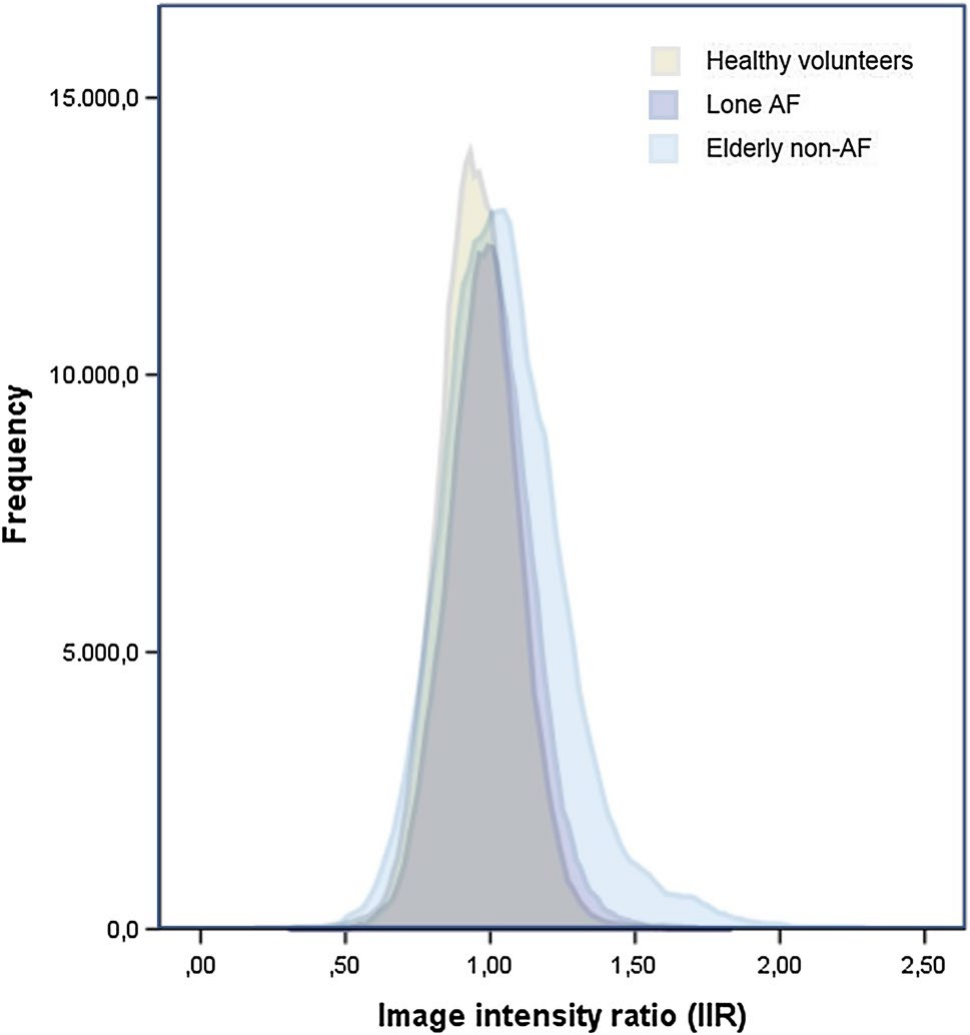
Histograms of image intensity ratios of the three different included groups. Notice the increasing right-sided skewness of lone AF and elderly non-AF. *AF* atrial fibrillation

In total, 308,877 pixel points were registered from healthy controls, 277,666 from lone AF patients and 314,393 from elderly non-AF patients and plotted as histograms (see Fig. 2).

Image intensity (IIR) threshold was extracted from healthy volunteer data (IIR mean + 2 SD) which was 1.21. When separating the healthy controls into an older and a younger fraction according to mean age, the IIR for < 37 (n = 6) and > 37 years (n = 5) were 1.22 ± 0.07 versus 1.21 ± 0.06 (p = 0.92).

The small deviation from the threshold set by Benito et al. [25] (1.20 vs. 1.21) was considered insignificant and hence for further analyses we performed the analyses with the IIR threshold value of 1.20.

Histograms of IIR-values for the three different groups showed skewness values of 0.16 for healthy controls, 0.20 for lone AF patients and 0.70 for elderly non-AF patients, representing increasingly right-tailed distributions.

### Detection of different degrees of native fibrosis

Subsequent analyses of the degree of fibrosis in the three groups with the IIR threshold of 1.20 revealed significantly different degrees of fibrosis with a median in healthy volunteers of 2.8% (1.3–8.3), lone-AF patients at 9.0% (3.9–12.0) and reaching the highest median value in elderly non-AF patients at 20.1% (10.2–35.8) (see Table 2). Table 2 shows conventional CMR variables. All measured CMR parameters were statistically similar between the three groups except for IIR-values and the degree of fibrosis.

**Table 2:** Cardiovascular magnetic resonance data

	Healthy volunteers n = 11	Lone AF n = 11	Elderly non-AF n = 11	*P* value
LV EF (%)	62.8 ± 5.4	61.0 ± 3.5	63.5 ± 12.2	0.74
LVEDVi	96.8 ± 11.2	96.7 ± 11.6	87.0 ± 19.1	0.20
LVESVi	36.3 ± 8.0	37.7 ± 5.5	33.5 ± 19.9	0.74
LA max volume indexed	57.6 ± 9.5	52.6 ± 11.2	54.2 ± 8.7	0.48
LA min volume indexed	24.9 ± 5.3	22.4 ± 5.6	26.9 ± 5.9	0.18
Left atrial late gadolinium enhancement				
Mean IIR	0.96 ± 0.13	1.00 ± 0.14	1.05 ± 0.21	<0.0001
Degree of fibrosis (IIR > 1.2)	2.8% (1.3–8.3)	9.0% (3.9–12.0)	20.1% (10.2–35.8)	0.001
Skewness	0.16	0.20	0.70	

*LV* left ventricle, *EF* ejection fraction, *EDVi* indexed end-diastolic volume, *ESVi* indexed end-systolic volume, *LA* left atrium, *IIR* image intensity ratio

Values are given as mean ± 1 SD or median (interquartile range)

Inter- and intra-observer agreement was excellent (inter-observer ICC = 0.957 (95% CI 0.880, 0.985) and intra-observer ICC = 0.995 (95% CI (0.986, 0.998). Bland–Altman plots are depicted in Fig. S1. Mean difference for inter-observer agreement was − 1.7 limits of agreement (− 10.25, 6.8) and for intra-observer − 1.0 (− 3.8, 1,7).

## Discussion

The present study verifies the use of the image intensity ratio (IIR) threshold value of 1.20 distinguishing healthy from fibrotic atrial myocardium in 1.5T LA LGE scans. This threshold value has previously been established in 3T scans on healthy volunteers [25] but never in 1.5T scans. When using this threshold in analyses of two different patient groups and healthy volunteers we found that young lone-AF patients with paroxysmal AF had increased degrees of atrial fibrosis compared to healthy volunteers in the same age-range. A group of elderly patients with no history of AF but various comorbidities that increases the risk of AF, revealed significantly increased degrees of atrial fibrosis compared to both other groups. This validation means that CMR studies of LA LGE performed on both 3T and 1.5T scanners can be analysed with the same threshold. This is also in line with the results by Chubb et al. [6] that LA LGE results in 1.5T and 3T are comparable.

The IIR analysis method was initially introduced by Khurram et al., who defined two IIR thresholds based on electroanatomic mapping (EAM): One at 0.97 distinguishing healthy myocardium from diffuse fibrosis and the other at 1.61 distinguishing diffuse fibrosis from dense fibrosis [23]. These thresholds were since adjusted by the same group to one IIR threshold of 1.2 based on EAM-data distinguishing fibrotic from healthy atrial wall [29, 30] with reference to a histopathological study by Harrison et al. suggesting new voltage thresholds for EAM [22]. These studies did not include any healthy volunteers and hence only rely on patients with pathologic atrial conditions but agree remarkably with the results from the present study.

CMR determination of LA LGE seems very promising [4, 8, 31, 32]. However, there a differing reports from different centres [5, 33], suggesting that CMR imaging of LA LGE needs to be streamlined with regards to image acquisition and analysis method. More widespread use of LA LGE, in research as well as clinically, depends on optimization of imaging parameters [6, 34] and objectification of imaging analyses [5, 12, 22, 23, 25, 35]. Consensus on imaging parameters and analysis method would make results of clinical studies more robust. Once there is a general agreement on how to perform LA LGE, results from different studies can be pooled and provide better foundation for clinical work [21].

Increased degree of native pre-ablation atrial fibrosis has been associated with age [9, 12, 32, 36], higher CHADS-score [12], hypertension [4, 12], stroke [24, 31], persistent AF [18, 20, 26, 36], re-entrant activity [8] and increased recurrence rates after ablation [4, 8, 10–13, 37]. The suggested associations between atrial fibrosis and age as well as with various comorbidities is continuously debated, since other studies have not found associations [20, 30, 38, 39].

Our results show that patients with lone AF have higher degrees of atrial fibrosis compared to age-matched healthy volunteers. These patients have no detectable structural heart disease and all included patients had paroxysmal AF, possibly the form with the least electrical and structural remodelling, which was also reflected by similar findings in other CMR parameters. It should especially be noted that atrial dimensions were similar in all three groups. This suggests that AF itself is associated with increased degree of fibrosis independently of structural remodelling, which is also supported by other studies [11, 25, 36, 39]. The question remains though, whether the fibrosis is the cause of AF or vice versa. In an elderly patient group with no history of AF but varying presence of diabetes mellitus, hypertension, congestive heart disease and/or previous stroke we found significantly higher degrees of fibrosis compared to both young groups.

The fibrotic atrial cardiomyopathy (FACM) concept of Kottkamp [38] explains the increased degrees of atrial fibrosis in lone AF patients but although studies are scarce, there is evidence that the degrees of fibrosis increase with age and presence of structural heart disease in patients without AF [36] and it may be that there are several different mechanisms that result in atrial fibrosis. The existence of a FACM syndrome does not rule out that diffuse atrial fibrosis can develop over time and possibly be augmented by various comorbidities. The question remains whether patients with increased degrees of fibrosis of any source are more likely to develop AF compared to patients without fibrosis.

### Study limitations

The study is based on a relatively small number of patients and healthy volunteers but nevertheless provides a reproduction of the same IIR threshold value as found by Benito et al. [25]. In general, the IIR method has not been validated against tissue samples but only EAM. While it would have strengthened the results with a further EAM-validation, this would not have been ethically responsible since the study was performed on healthy volunteers.

Fibrosis is a dynamic process stretching from initial inflammation and ultimately potentially development of scar, i.e. stable fibrotic tissue. As in all LGE studies, there is the possibility that visualised LGE in fact reflects early inflammatory processes/oedema in the myocardium and not stable fibrotic tissue [40], hence enhancement may instead be termed suspected fibrosis.

We decided to only test the threshold distinguishing between healthy and fibrotic atrial wall since this separation is clinically relevant and we have found no studies suggesting further value of two thresholds.

The thickness of the atrial wall is close to the resolution of CMR and hence especially for thin in-plane structures the 3D-acquisition can provide low accuracy and some degree of partial volume effects may be unavoidable.

While some studies find no correlation between age and increased fibrosis [8, 20, 26, 38] and others do [9, 12], we decided to set an upper age-limit at 45 years to avoid possible age-related fibrosis in healthy volunteers.

With regards, to the elderly non-AF population, there is the possibility that they have experienced asymptomatic AF episodes.

## Conclusion

In 1.5T LA LGE scans on healthy volunteers we found an IIR threshold of 1.21 for healthy versus fibrotic tissue which is similar to the previously found threshold of 1.20 in 3T scans. The IIR threshold between healthy and fibrotic atrial myocardium of 1.2 can thus be used for both field strengths.

Using the IIR threshold of 1.20, we found increased degrees of left atrial fibrosis in patients with lone paroxysmal AF compared to healthy controls in the same age-range. Elderly patients with no known history of AF but known diabetes mellitus, hypertension, congestive heart disease and/or previous stroke had significantly higher degrees of left atrial fibrosis than both groups of younger individuals.

## Electronic supplementary material

Below is the link to the electronic supplementary material.

Supplementary material 1 (DOCX 17 kb)
