# Supplementary material for: Verification of threshold for image intensity ratio analyses of late gadolinium enhancement magnetic resonance imaging of left atrial fibrosis in 1.5T scans
Source: Int J Cardiovasc Imaging. 2019 Nov 20;36(3):513–20. doi: 10.1007/s10554-019-01728-0 (PMC7080681; doi:10.1007/s10554-019-01728-0)
Supplement: Supplementary file 1 — Supplementary material 1 (DOCX 17 kb) [file 10554_2019_1728_MOESM1_ESM.docx]

| 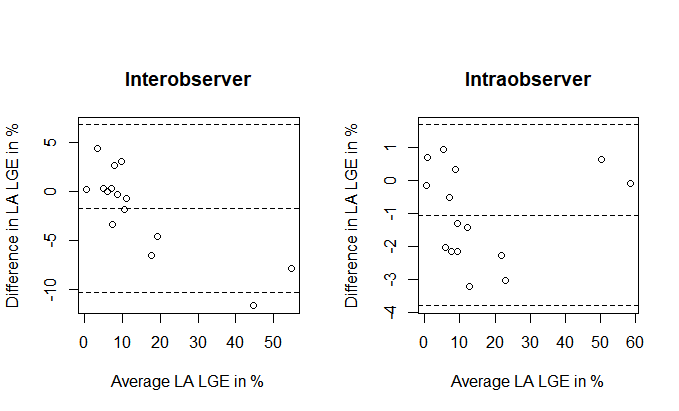 |
| --- |
| **Figure S1** Bland-Altman plots of inter- and intra-observer agreement. The lines represent the mean difference and the 95% limits of agreement.  LA LGE = Left atrial late gadolinium enhancement |
